# Vestibular System and Hearing Involvement in Patients with Turner Syndrome

**DOI:** 10.3390/jcm15062392

**Published:** 2026-03-20

**Authors:** Victoria Díaz Sánchez, Helena España Dos Santos, Luis Cabrera Pérez, Susana Marcos Alonso, Fernando Benito González, Hortensia Sánchez Gómez, Ana Belen Alonso San Eloy, Mercedes Cecilio Rivas, Ángel Batuecas Caletrio

**Affiliations:** Otoneurology Unit, ENT Department, University Hospital of Salamanca, Instituto de Investigación Biomédica de Salamanca (IBSAL), University of Salamanca, 37007 Salamanca, Spain; vdiazsan@saludcastillayleon.es (V.D.S.); hespana@saludcastillayleon.es (H.E.D.S.); lcabrerap@saludcastillayleon.es (L.C.P.); fbenitogo@saludcastillayleon.es (F.B.G.); hortensiasanchez@saludcastillayleon.es (H.S.G.); abalonso@saludcastillayleon.es (A.B.A.S.E.); mcecilio@saludcastillayleon.es (M.C.R.); abatuecas@saludcastillayleon.es (Á.B.C.)

**Keywords:** Turner syndrome, vestibular system, video head impulse test, computerized dynamic posturography

## Abstract

**Background**: Turner syndrome is a genotypic disorder in females characterized by the total or partial absence of an X chromosome. While cardiovascular issues and sensorineural hearing loss are well-documented, vestibular system involvement remains understudied. This study aims to examine vestibular system involvement in patients with Turner syndrome and assess if they exhibit a higher prevalence of peripheral vestibular pathology compared to the general population. **Methods**: A retrospective longitudinal study was conducted with 21 Turner syndrome patients and 21 age-matched controls. Evaluations included clinical history, otoscopy, pure tone audiometry, the Video Head Impulse Test (vHIT) to measure vestibulo-ocular reflex gain, and computerized dynamic posturography, specifically the Sensory Organization Test (SOT) and Stability Limits Analysis. **Results**: Turner syndrome patients showed significantly higher hearing thresholds across all frequencies compared to controls (*p* < 0.001). In the vHIT, 30% of the Turner group presented pathological results, with significant gain reductions in the right horizontal and left posterior semicircular canals. Posturography revealed a significant reduction in overall stability (*p* = 0.006) and a significantly lower vestibular index (*p* = 0.011) in the Turner group. Additionally, patients with Turner syndrome demonstrated significant impairments in directional control, reaction time, and excursion points during Stability Limits Analysis. **Conclusions**: Patients with Turner syndrome are more likely to experience vestibular disorders, a finding likely associated with estrogen deficiency and the loss of its protective effect on the inner ear. These results highlight the necessity of including vestibular and posturographic assessments in the routine clinical follow-up of these patients to facilitate early detection and rehabilitation, even in the absence of overt symptoms like vertigo.

## 1. Introduction

Turner syndrome is a genotypic disorder that affects only females, associated with the absence of one of the sex chromosomes [1]. The X chromosome defect may be total, with the typical karyotype 45X0, or partial, presenting structural abnormalities of the X chromosome (isochromosome, ring chromosome, etc.) [2]. The severity of clinical characteristics appears to correlate with the extent of X chromosome material loss [3]. In recent years, numerous studies have been conducted to explain the identification of genetic and epigenetic changes in patients with Turner syndrome [4].

This syndrome affects approximately 1 in 2500 live births, although most affected fetuses are miscarried [5]. There are certain signs that may lead to a suspected diagnosis of Turner syndrome during pregnancy, mainly intrauterine growth retardation, with this stage being the first diagnostic peak. At birth, babies commonly measure about 2–3 cm less than estimated for gestational age and are about 500 g lighter. It is common to see a winged neck (pterygium colli, from hypoplasia of the lymphatic vessels in the cervical region), low-set ears and hairline, and other craniofacial malformations, widely spaced nipples, and an arched palate from the outset.

In childhood, short stature is the most frequent and consistent finding. The so-called “Turner phenotype” becomes more noticeable with growth, presenting a triangular facies, micrognathism, epicanthic folds, and hypertelorism. Pectus excavatum, separated nipples, cubitus valgus, shortened metacarpals/metatarsals, hypoplastic nails, and lymphedema are common [1,6]. In addition, patients with Turner syndrome have estrogen deficiency due to premature ovarian failure. This leads to amenorrhea and, therefore, infertility, without the development of female secondary sexual characteristics. This stage is the second peak of diagnosis, between the ages of 5 and 20, and it is important to make a differential diagnosis aimed at addressing the different manifestations through a multidisciplinary approach, with the participation of different specialists [7,8].

In adulthood, cardiovascular morbidity is the most important factor to consider in patients with Turner syndrome. There is a higher prevalence of bicuspid aortic valve (25%), aortic arch elongation (40–50%), aortic dilation (3–42%), and other congenital heart diseases [9]. These patients also present with renal malformations, immune system disorders, endocrine disorders, and ENT problems [10]. Among the latter, it is worth noting a higher prevalence of recurrent acute otitis media between the ages of 1 and 6 and a higher prevalence of cholesteatomas, which contribute to conductive hearing loss, with a prevalence of 10 to 40% in women with Turner syndrome. In addition, approximately half of these patients develop sensorineural hearing loss that appears in early life and may be associated with estrogen deficiency. It has been hypothesized that sensorineural hearing loss could be associated with vestibular system dysfunction, as the vestibular system and the cochlea have a common embryonic origin. Given that 52% of children with genetic hearing loss have peripheral vestibular dysfunction and up to 79% of patients with bilateral vestibulopathy have hearing loss, it is plausible that vestibular disorders may also occur in Turner syndrome [11,12]. However, vestibular disorders have not been sufficiently studied and there is little literature on this subject. Vertigo symptoms significantly impact quality of life, with 86.4% of patients experiencing deterioration and 42.3% developing depressive symptoms. Given this considerable functional and psychological impact, it is essential to further study vestibular dysfunction as a comorbidity in Turner syndrome [13].

The objective of this study is to examine vestibular system involvement in patients with Turner syndrome and to assess whether they show a higher prevalence of peripheral vestibular pathology than the general population.

## 2. Materials and Methods

A retrospective longitudinal study was conducted by comparing two groups: patients diagnosed with Turner syndrome and a control group. Twenty-one patients diagnosed with Turner syndrome belonging to the Alejandra Turner Syndrome Association in Salamanca who attended consultations at the Otoneurology Unit of the Salamanca University Hospital were recruited. Inclusion criteria were established, consisting of a confirmed diagnosis of Turner syndrome in the case group and an age range between 6 and 60 years. Patient recruitment took place between June 2024 and June 2025. Exclusion criteria were neurological or psychiatric conditions that could interfere with vestibular testing or the inability to complete the examination; systemic or hormonal disorders (other than those intrinsic to Turner syndrome) that might affect vestibular function; or use of medications that could impact the central nervous system or vestibular stability.

The control group consisted of 21 healthy female participants recruited during the same period as the study group to ensure a consistent and objective screening process. To minimize confounding variables, the control group was age-matched to the Turner syndrome group. Inclusion in the control group required thorough screening to exclude any factors that might impact the results. Candidates were excluded if they had a history of vestibular pathology, dizziness, or instability; sensorineural hearing loss or tinnitus; a history of ototoxic medication use; or any neurological, psychiatric, hormonal, or systemic disorder consistent with the exclusion criteria applied to the study group.

All patients provided detailed medical histories and demographic and clinical variables were collected, such as age, height (cm), weight (kg), BMI (kg/m^2^), history of otitis, history of vestibular pathology, and hormone replacement therapy.

These patients underwent a systematic ENT physical examination:

### 2.1. Otoscopy

Otoscopy was classified as normal or pathological. Pathological otoscopy was defined as the presence of myringosclerosis, tympanic perforations, serous retratympanic effusion, transtympanic drainage, cholesteatoma, as well as retraction pockets or atelectasis of the tympanic membrane or neotympanum after tympanoplasty.

### 2.2. Audiometry

Pure air conduction tones were measured at frequencies of 250, 500, 1000, 2000, 3000, 4000 and 8000 hertz (Hz) and pure bone conduction tones at frequencies of 250, 500, 1000, 2000 and 4000 Hz, for both the right and left ears, and the pure tone average (PTA) calculated, an audiometric index that reflects the average of the hearing thresholds measured in decibels (dB) obtained at frequencies of 500, 1000, 2000 and 4000 Hz [14].

To perform the audiometry in this study, we used Clinical Audiometer AC40 (Interacoustics, Middelfart, Denmark).

### 2.3. Video Head Impulse Test (vHIT)

This test evaluates the vestibulo-ocular reflex (VOR), an involuntary mechanism that stabilizes the gaze when there is head movement, causing an eye movement opposite and equal in speed to that of the head, allowing images to remain fixed and stable on the retina. This reflex records the angular movement of the head in the three positions of space, detected by the six semicircular canals [14]. The stimulus frequency used during the examination exceeds 2.5 Hz, which is recognized as the physiological frequency range for the vestibular system [15].

We must place glasses with an accelerometer and a video camera on the patient. A reference point is placed, one meter away, so that the patient keeps their gaze fixed, and the doctor, behind the patient, holds their head while turning it quickly [14].

The head and eye movement velocities are measured and recorded. The relation between these two velocities is obtained from their ratio and is called VOR gain. The perfect gain is 1, which means that both movements have exact velocities, although in opposite directions. Gains below 0.8 are considered pathological in horizontal semicircular canal (HSC) and below 0.7 in the vertical canals, posterior (PSC) and superior (SSC). The presence of corrective saccades is also considered a pathological finding. Covert saccades occur during the head movement, whereas overt saccades appear after the head impulse has ended [14,16].

To perform the vHIT in this study, we used Otometrics ICS Impulse equipment (Otometrics, Taastrup, Denmark). To minimize the interobserver variability, all examinations were conducted by the same experienced investigator. For each participant, a minimum of 20 valid stimuli were recorded for each direction (right and left horizontal, superior, and posterior), ensuring that only high-quality impulses were included for analysis. Any stimuli rejected by the system due to artifacts, such as eye blinking or inadequate head velocity, were excluded. The variables recorded for each canal included the mean gain, the presence or absence of covert and overt saccades, saccades velocity and the PR index.

### 2.4. Computerized Dynamic Posturography

This is a technique that objectively assesses postural control and balance in different conditions by modifying the surface and visual environment. This test is performed using a posturograph, which consists of a platform capable of tilting in the same direction and angle as the patient’s sway; and a visual environment, which can remain fixed or be mobile, thus generating visual conflict [17,18].

The posturograph (Neurocom equitest system version 7.0) includes two main tests. The first is the Sensory Organization Test (SOT), which analyses the contribution of the vestibular, visual and somatosensory systems through six conditions in which visual information, somatosensation and platform stability are modified. Parameters such as the percentage of overall balance and reference patterns are obtained, from which various sensory quotients are compared to identify patterns of somatosensory, visual or vestibular deficits, obtaining objective data on the use of the information provided by each system (Table 1) [14,17].

The second test is the Stability Limits Analysis, which quantifies the patient’s ability to shift their center of gravity within a theoretical ellipse of stability. It assesses eight directions of movement and includes parameters such as reaction time, displacement speed, initial excursion, maximum excursion, and directional control. Altered values in these measures may reflect vestibular, somatosensory, neuromuscular dysfunctions, or difficulties in motor planning (Table 2) [16,18].

### 2.5. Statistical Analysis

This study incorporated descriptive and analytical components. The clinical characteristics and outcomes of the patients were summarized using descriptive statistics, expressing continuous variables as means and standard deviations, and categorical variables as frequencies and percentages.

Comparisons between the Turner and control groups were performed using Student’s *t*-test for independent samples in the case of continuous variables, and chi-square tests or Fisher’s exact test for categorical variables, as appropriate. A *p*-value < 0.05 was considered statistically significant. The normality of continuous variables was assessed using the Shapiro–Wilk test, and confirmed prior to analysis. All statistical analyses were performed using SPSS software, version 21 (IBM, Armonk, NY, USA).

Approval for this study was obtained from the hospital’s ethics committee through the Institutional Review Board (IRB) with code PI 2025 04 1898, on 2 June 2025.

## 3. Results

In terms of the descriptive analysis of the sample, all patients in both the case and control groups were female, and there were twenty-one patients in each group. Patients in the case group were age-matched with patients in the control group. The mean age was 24.95 years (standard deviation (SD) ± 16.83).

The average height was 1.47 m (SD ± 0.102) in the Turner group and 1.61 m (SD ± 1.124) in the control group. The average weight was 52.9 kg (kg) (SD ± 18.7) in the Turner group and 63.6 kg (SD ± 14.6) in the control group. No significant differences in weight (*p* = 0.157) were observed between groups; however, height was significantly lower in the Turner group (*p* = 0.004) compared to the control group. Regarding BMI values, the results in the Turner group were 25.0 (SD ± 6.20) and in the control group 23.7 (SD ± 2.89), with no statistically significant differences found (Table 3).

A percentage of 85.7% of Turner patients had a history of ear problems and 42.9% had hearing symptoms at the time of consultation, with significant differences (χ^2^ = 11.0; *p* < 0.001) compared to a minority proportion in the control group. Nineteen per cent (4 patients) of the Turner group had a history of vestibular pathology, three of them with benign paroxysmal positional vertigo and one requiring vestibular rehabilitation due to instability; significant differences were also found in vestibular pathological history (χ^2^ = 4.22; *p* = 0.040).

All patients with Turner syndrome, except one, were undergoing active hormone treatment at the time of consultation, compared to none of the patients in the control group, with statistically significant differences. Otoscopy was pathological in 52.4% of Turner patients and in 9.5% of control patients, showing variability in favor of patients with Turner syndrome, reaching significance (*p* < 0.05).

Regarding the hearing levels at specific frequencies, highly significant differences were found at all frequencies, in both the right and left ears (*p* < 0.001 at almost all frequencies) (Table 4).

The mean PTA values were 26.6 (SD ± 16.3) in the right ear and 28.4 (SD ± 17.4) in the left ear in Turner patients, while in control patients they were 6.77 ± 2.62 and 6.25 ± 1.52, respectively. Statistically significant results were obtained in favor of Turner patients (*p* < 0.01).

As for the results obtained in the VHIT, it was observed that the values of the patients in the Turner syndrome group were lower than those of the control group in most semicircular canals (Table 5). Statistically significant differences were observed in the gain of the right horizontal semicircular canal (t = 3.138; *p* = 0.003) and in the gain of the left posterior semicircular canal (t = −2.511; *p* = 0.016) between the gain values of the Turner syndrome group and the control group. It should be noted that one patient presented with areflexia of both posterior semicircular canals. The rest of the semicircular canals showed normal or slightly lower values without reaching statistical significance. The statistical test used was Fisher’s exact test.

In terms of saccades, in the case group, 6 patients had covert saccades and 6 patients had overt saccades, whereas in the control group, no patients had covert or overt saccades. The saccades did not show significant differences in any semicircular canals between the groups.

In total, 10 pathological VHITs were identified. Ten semicircular canals with low gains were observed in a total of six patients, and nine patients presented corrective saccades, compared to no pathological VHITs in the control group.

**Figure 1:**
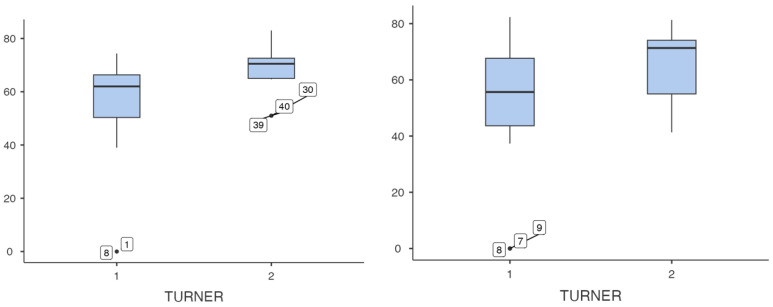
Box plots showing the distribution of conditions 5 (**left**) and 6 (**right**). On the *x*-axis, group 1 corresponds to Turner syndrome patients and group 2 to control patients. The *y*-axis represents the values of each condition. (condition 5: *p* = 0.011; condition 6: *p* = 0.009; performed using student’s *t*-test).

Regarding the sensory organization test of dynamic posturography, patients with Turner syndrome showed a significant reduction in overall stability. The overall balance result was 71.7 ± 9.60% in the Turner group and 79.3 ± 6.57% in the controls, with a statistically significant difference (*p* = 0.006). In the analysis of the SOT conditions, statistically significant results were obtained in conditions 5 and 6, showing greater instability in the Turner group (condition 5: 50.0 ± 20.6% in the Turner group and 68.3 ± 8.85% in the control group; *p* = 0.011; condition 6: 50.2 ± 23.9 in the Turner group and 66.3 ± 11.1% in the control group; *p* = 0.009). In the rest of the conditions, no significant results were found, but there was a trend towards significance (Table 6 and Figure 1).

**Figure 2:**
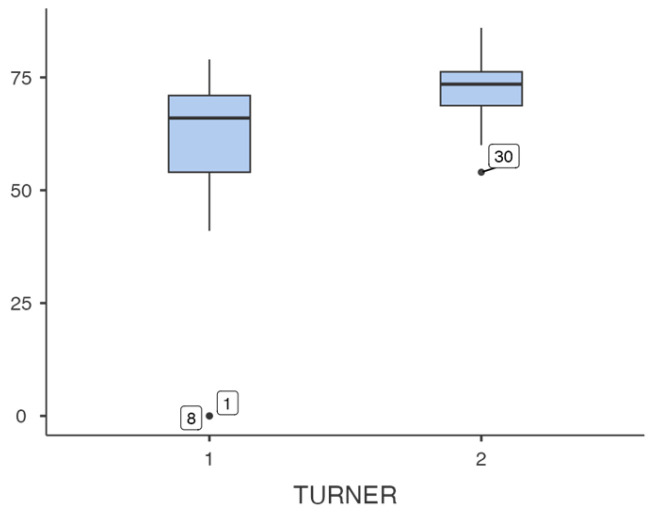
Box plots showing the vestibular index. On the *x*-axis, group 1 corresponds to Turner syndrome patients and group 2 to control patients. The *y*-axis represents the values of vestibular index. Between-group differences were significant (*p* = 0.011; performed using student’s *t*-test).

In terms of sensory indices, the mean vestibular index score was 58.9 ± 21.8 in the Turner group and 72.8 ± 8.04 in the control group, with statistically significant differences (*p* = 0.011) (Table 7 and Figure 2).

In the analysis of stability limits, there are significant results in directional control (74.8 ± 8.55% in the Turner group and 81.3 ± 5.48% in the control group; *p* = 0.006), reaction time (0.364 ± 0.162 s in the Turner group and 0.857 ± 0.163 s in the control group; *p* = 0.016), initial point of excursion (%) (80.9 ± 8.77% in the Turner group and 87.0 ± 5.77% in the control group; *p* = 0.014) and maximum excursion point (93.4 ± 4.62% in the Turner group and 97.3 ± 4.08% in the control group; *p* = 0.006) (Table 8).

## 4. Discussion

Estrogens serve as indispensable homeostatic modulators of the inner ear’s microenvironment by binding to ligand-activated nuclear receptors, called alpha and beta (ERα and ERβ). These receptors transcriptionally regulate essential potassium ion transporter proteins, specifically KCNE1, KCNQ1, and ATP1B2, within the stria vascularis, which are fundamental for maintaining the electrochemical gradients and endolymphatic potential required for signal transduction [18].

The presence of these receptors has been demonstrated in the inner ear of mice and humans, suggesting the direct action of this hormone on auditory and vestibular function. Patients with Turner syndrome, who have estrogen deficiency secondary to ovarian dysgenesis, have a higher prevalence of sensorineural hearing loss, as demonstrated in medical literature. Recent studies suggest that the presence of estrogens is considered a protection against both conductive and sensorineural hearing loss [19,20]. In addition, there is a study in which a mouse model with Turner syndrome (X0 mouse), called the “Turner mouse”, has been created, in which the hearing impairments observed in Turner syndrome are reproduced. Furthermore, the same pattern of estrogen receptor expression has been demonstrated in healthy mice [20].

This study characterizes significant divergences in the otological and neurotological profiles of patients with Turner syndrome compared to age-matched controls. Turner patients exhibited a markedly elevated prevalence of otologic history (85.7%) and prior vestibular pathology (19%). Comprehensive audiometric assessments demonstrated significantly attenuated hearing thresholds across the entire frequency spectrum (*p* < 0.001).

Regarding the vHIT, statistically lower gains were identified in the right horizontal and left posterior canals (*p* < 0.05). Furthermore, a higher incidence of pathological corrective saccades was observed in the Turner syndrome group, affecting 30% of the subjects.

Moreover, the most significant findings emerged from Computerized Dynamic Posturography, which revealed impaired postural control and a significant decrement in overall stability (*p* = 0.006). Turner syndrome patients demonstrated markedly reduced scores in Sensory Organization Test conditions 5 and 6, resulting in a significantly diminished vestibular sensory index (*p* = 0.011). Additionally, Stability Limits Analysis indicated significant impairments in directional control, reaction time, and maximum excursion.

These data suggest that patients with Turner syndrome exhibit a functional deficiency in the integration of vestibular information for balance maintenance, likely associated with the loss of the protective effects of estrogens on the inner ear.

However, there is very little literature establishing or demonstrating a clear correlation between vestibular dysfunction and Turner syndrome. We highlight the article by Wahlberg et al. (2013) [21], which investigates the balance of patients with Turner syndrome using dynamic posturography. Nineteen women with this condition and 19 control patients, matched for age, were studied. The posturography results revealed that patients with Turner syndrome did not show statistically significant differences from the control patients, but they did show worse results. However, in this study, the authors report that the altered results are related to an impairment in dynamic balance skills, caused by a combination of central involvement in the cerebellar regions, probable damage to the inner ear, and involvement of the tendons, muscle tone, and proprioception in patients with Turner syndrome, without establishing a direct causality with specific peripheral vestibular damage.

As for the results of our study, in the sensory organization test, the results indicate an overall reduction in postural stability in patients with Turner syndrome. The alterations in conditions 5 and 6 show a worse score in conditions that require predominance of the vestibular system. In the sensory indices, the vestibular index was the only one with a significant difference, suggesting a functional vestibular alteration in patients with Turner syndrome. Furthermore, in the analysis of stability limits, alterations in postural control are evident in patients with Turner syndrome with clinically relevant results that, with a larger sample size, could be significant. However, the connective tissue involvement inherent to Turner syndrome deserves consideration. Haploinsufficiency of the SHOX gene, a transcription factor that serves as a master regulator of mesodermal tissue development, results in generalized growth disturbances extending to the musculoskeletal system, including short stature, cubitus valgus, and altered bone geometry. Accordingly, this musculoskeletal involvement should be taken into account when interpreting the study results [22].

We would also like to highlight the article by Baxter et al. (2014) [19], in which they report an isolated case of bilateral vestibular dysfunction in a patient with Turner syndrome, as determined by videonystagmography, revealing a bilateral reduction in VOR gain values in the HSC. This is the first article to refer to an impairment of the vestibular system in this study population. The patient, who was completely asymptomatic from a vestibular point of view, showed low gain in the horizontal semicircular canals (0.29 right and 0.36 left) when undergoing VHIT. Until this publication, it had not been demonstrated that patients with Turner syndrome could have vestibular system involvement and that this involvement could be associated with estrogen deficiency.

Compared to our study, the results obtained in the VHIT show a significant reduction in vestibulo-ocular reflex gain in the left posterior and right horizontal semicircular canals in patients with Turner syndrome, compared to healthy controls. This decrease in gain is accompanied by a higher proportion of corrective saccades, both covert and overt, in the Turner group. In total, 10 pathological VHITs were identified, 47.62% of patients with Turner syndrome. After an exhaustive review of the literature, some studies suggest the existence of vestibular involvement in Turner syndrome; however, no larger series including objective vestibular tests demonstrating such involvement have been found. These findings suggest that patients with Turner syndrome may be associated with vestibular hypofunction.

As for the limitations of our study, more powerful studies with larger sample sizes are needed to obtain more definitive results regarding vestibular involvement in Turner syndrome. It should also be noted that our study did not distinguish subgroups according to karyotype (45X, mosaic, isochromosome, ring X chromosome, etc.). Moreover, in the study we did not include other tests such as caloric testing, cervical VEMPs and ocular VEMPs. This will be considered in future research lines. Another limitation concerns the inclusion of otoacoustic emissions and auditory brainstems’ response audiometry in future studies to provide information on cochlear function and neural auditory pathways in patients with Turner syndrome.

## 5. Conclusions

In conclusion, after analyzing the data collected, we could suggest that patients with Turner syndrome are more likely to have vestibular disorders, probably associated with estrogen deficiency. Although there is a well-documented relationship between Turner syndrome and sensorineural hearing loss, no previous conclusive studies have established an association between Turner syndrome and vestibular dysfunction. The pathophysiological mechanism could be related to the protective effect of estrogens in the inner ear, both in the cochlea and in the vestibular system. This finding highlights the importance of incorporating vestibular and posturographic assessment into the routine clinical follow-up of these patients, even in the absence of symptoms of vertigo or instability, in order to detect such possible sensory alterations early on and establish prevention measures and treatments with vestibular rehabilitation.

## Figures and Tables

**Table 1:** Description of the six conditions evaluated in the Sensory Organization Test (SOT). Each condition varies in the sensory information used, thus analyzing the visual, vestibular and somatosensory systems in maintaining balance [14].

Condition	Situation	Normal Value
Condition 1	Eyes open, fixed visual environment and fixed platform.	Above 90%
Condition 2	Eyes closed and fixed platform.	85–90%
Condition 3	Eyes open, moving visual environment and fixed platform.	80–85%
Condition 4	Eyes open, fixed visual environment and moving platform.	70–80%
Condition 5	Eyes closed and mobile platform.	70–75%
Condition 6	Eyes open, mobile visual environment and mobile platform.	60–75%

**Table 2:** Description of the parameters evaluated in the Stability Limits Analysis.

Stability Limits Analysis Parameters	Normal Value
Reaction time	0.6–1.0 s
Displacement speed	2.5–4.0°/s
Initial excursion	80–90%
Maximum excursion	~100%
Directional control	85–100%

**Table 3:** Demographic and anthropometric characteristics of the Turner syndrome and control groups. Values are expressed as mean ± standard deviation. Statistical comparisons were performed using [Student’s *t*-test/Mann–Whitney U test]. Significance was set at *p* < 0.05. The calculation was performed using student’s *t*-test.

	Turner	Control	*p*
Age (years)	24.95 ± 16.83	24.95 ± 16.83	
Height (m)	1.47 ± 0.10	1.61 ± 1.124	0.004
Weight (kg)	52.9 ± 18.7	63.6 ± 14.6	0.157
BMI	25.0 ± 6.2	23.7 ± 2.89	0.61

**Table 4:** Results of air conduction and bone conduction audiometry in both ears, comparing the Turner group and the control group. Values correspond to mean ± standard deviation (dB) for each frequency evaluated. *p*-values corresponding to the comparison between groups are included, with *p* < 0.05 considered statistically significant, emphasized in bold type. The calculation was performed using student’s *t*-test.

Audiometry	Right Ear			Left Ear		
	Turner	Control	*p*	Turner	Control	*p*
Air Conduction (dB)						
250 Hz	20.5 ± 12.0	6.50 ± 3.7	**<0.001**	22.1 ± 13.6	8.25 ± 4.1	**<0.001**
500 Hz	23.1 ± 13.3	8.00 ± 3	**<0.001**	24.3 ± 13.5	7.75 ± 3.4	**<0.001**
1000 Hz	25.2 ± 17.1	7.50 ± 3.0	**<0.001**	26.9 ± 19.3	6.00 ± 2.6	**<0.001**
2000 Hz	27.6 ± 18.9	5.00 ± 2.6	**<0.001**	29.5 ± 19.2	3.75 ± 2.8	**<0.001**
3000 Hz	29.8 ± 20.5	7.75 ± 5.7	**<0.001**	31.7 ± 21.6	8.25 ± 4.9	**<0.001**
4000 Hz	30.0 ± 21.9	7.00 ± 5.7	**<0.001**	32.6 ± 23.2	7.50 ± 4.1	**<0.001**
8000 Hz	39.3 ± 23.9	14.8 ± 12.8	**<0.001**	41.4 ± 25.2	14.8 ± 9.7	**<0.001**
Bone Conduction (dB)						
250 Hz	10.7 ± 11.8	2.00 ± 2.5	**0.002**	6.61 ± 10.4	3.50 ± 4.8	**0.044**
500 Hz	16.0 ± 13.1	4.25 ± 4.7	**<0.001**	14.3 ± 12.6	4.25 ± 3.7	**0.001**
1000 Hz	19.0 ± 16.5	3.75 ± 3.9	**<0.001**	17.6 ± 14.8	3.00 ± 2.0	**<0.001**
2000 Hz	24.8 ± 18.7	3.00 ± 3	**<0.001**	24.5 ± 18.6	3.75 ± 4.6	**<0.001**
4000 Hz	23.3 ± 18.9	3.75 ± 4.6	**<0.001**	21.7 ± 17.8	4.00 ± 4.5	**<0.001**

**Table 5:** Results of the video head impulse test (vHIT) in the horizontal (HSC), posterior (PSC) and superior (SSC) semicircular canals for both ears in the Turner group and the control group.

Patients	Pathological VHIT
Patient number 1	Right ear: HSC overt saccade.
Patient number 2	Left ear: HSC overt saccade. Left ear: PSC overt saccade Left ear PSC gain: 0.47.
Patient number 3	Right ear: PSC covert saccade.
Patient number 7	Left ear: HSC overt saccade. Left ear: SSC covert saccade.
Patient number 8	Right ear: HSC overt saccade. Right ear PSC gain: 0.66. Left ear: HSC overt saccade. Left ear PSC gain: 0.68.
Patient number 9	Right ear: PSC overt saccade.
Patient number 10	Right ear: HSC covert saccade. Right ear SSC gain: 0.69. Left ear HSC gain: 0.78. Left ear: HSC covert saccade.
Patient number 11	Right ear HSC gain: 0.55. Right ear: HSC covert saccade.
Patient number 13	Right ear PSC gain: 0.67. Left ear HSC gain: 0.58.
Patient number 18	Right ear PSC gain: 0. Right ear: PSC covert saccade. Left ear PSC gain: 0. Left ear: PSC covert saccade.

**Table 6:** Sensory Organization Test (SOT) results for the six conditions, expressed as a percentage (%), comparing the Turner group with the control group. Values are presented as mean ± standard deviation, together with the *p*-value corresponding to the comparison between groups. A *p*-value < 0.05 was considered statistically significant, emphasized in bold type. The calculation was performed using student’s *t*-test.

SOT (%)	Turner Syndrome Group	Control Group	*p*-Value
Condition 1	92.9 ± 2.45	90.7 ± 3.21	0.339
Condition 2	69.6 ± 7.21	90.4 ± 4.33	0.678
Condition 3	90.0 ± 2.66	91.0 ± 3.59	0.356
Condition 4	77.8 ± 7.59	82.2 ± 8.66	0.092
Condition 5	55.0 ± 20.6	68.3 ± 8.85	**0.011**
Condition 6	50.2 ± 23.9	66.3 ± 11.1	**0.009**

**Table 7:** Somatosensory, visual and vestibular indices obtained in the Sensory Organization Test (SOT), comparing the Turner group and the control group. Values are expressed as mean ± standard deviation, together with the *p*-value corresponding to the comparison between groups. A *p*-value < 0.05 was considered statistically significant, emphasized in bold type. The calculation was performed using student’s *t*-test.

SOT	Turner Syndrome Group	Control Group	*p*-Value
Somatosensory index	98.0 ± 2.01	96.7 ± 2.54	0.076
Visual index	83.9 ± 7.04	87.5 ± 7.07	0.102
Vestibular index	58.9 ± 21.8	72.8 ± 8.04	**0.011**

**Table 8:** Results of the Stability Limits Analysis, comparing the Turner group with the control group. Values are presented as mean ± standard deviation, together with the *p*-value corresponding to the comparison between groups. A *p*-value < 0.05 was considered statistically significant, emphasized in bold type. The calculation was performed using student’s *t*-test.

Analysis of Stability Limits	Turner Syndrome Group	Control Group	*p*-Value
Reaction time (s)	0.364 ± 0.162	0.857 ± 0.163	**0.016**
Movement speed (°/s)	4.87 ± 1.36	4.36 ± 0.921	0.164
Initial excursion point (%)	80.0 ± 8.77	87.0 ± 5.77	**0.014**
Point of maximum excursion (%)	93.4 ± 4.62	97.3 ± 4.08	**0.006**
Directional control (%)	74.8 ± 8.55	81.3 ± 5.48	**0.006**

## Data Availability

The data supporting the findings of this study are available within the article.
